# The color red distorts time perception for men, but not for women

**DOI:** 10.1038/srep05899

**Published:** 2014-07-31

**Authors:** Masahiro Shibasaki, Nobuo Masataka

**Affiliations:** 1 Primate Research Institute, Kyoto University, Inuyama, Japan

## Abstract

We investigated the effect of the color red on time perception using a temporal bisection task with human adults. The results showed that the perceived duration of a red screen was longer than was that of a blue screen. However, the results reflected sex differences; men, but not women, overestimated the duration of the red screen. Additionally, the reaction times to a red screen were faster than those to a blue screen, and we found a significant correlation between reaction time and the tendency to overestimate the duration of a red screen. Participants who reacted quickly to a red screen overestimated its duration. These results are discussed within the context of recent studies indicating that the color red exerts certain special psychological effects on human behavior.

It is well known that colors affect our behavior and emotions. For example, long-wavelength colors, such as red, are often referred to as exciting, whereas short-wavelength colors, such as blue, are considered relaxing. These assumptions have been confirmed in studies using physiological measures of excitation and arousal. Ali^1^ directed a red or blue light directly into the eyes of the participant through a projector for 10 min. The EEG results showed a faster recovery of *alpha* waves under the blue than under the red condition. Jacobs and Hustmyer^2^ used galvanic skin response (GSR) as an indicator of skin conductance and found a higher level of conductance when a red screen than when a blue one was presented.

Recent studies have shown that observing emotional expressions affects subjective experiences of time by increasing the level of arousal of the perceiver^3^,^4^,^5^. Accordingly, arousal in the presence of a red light may be associated with distorted perceptions of time. However, few studies regarding the effects of color on time perception have been conducted, and Smets^6^ was the first to suggest a possible relationship between these phenomena. Smets^6^ exposed subjects to red and then blue light for 45 s each. After exposure to both colors, subjects were asked to estimate the length of time that each color had been presented. Subjects perceived the time interval spent in the presence of red light as shorter than the same time interval spent in the presence of the blue light. Smets^6^ concluded that viewing red accelerated the passage of subjective time compared with viewing blue. However, if red accelerates the passage of subjective time, the duration of the exposure to red should be perceived as longer than that of the exposure to blue. Caldwell and Jones^7^ asked subjects to estimate the passage of 35 s and 45 s in the presence of red, white, and blue light using the method of production. The results indicated that color did not affect the estimates of time intervals, raising questions about the assumption that “warm” colors are more arousing than are “cool” colors. Gorn et al.^8^ investigated the effect of web page color on the perceived rapidity of a download. The results suggested a significant effect of color on perceived rapidity, with participants under the blue condition perceiving the page as downloading faster than participants under the red condition.

As these studies indicate, the effects of color on time perception are not consistent. Thus, the present study investigated the influence of color on time perception using a temporal bisection procedure that has been well established in the study of interval timing. We compared the effects of red with those of blue because blue is the most commonly selected color in studies of color's effects upon human behavior and emotions.

## Results

**Figure 1:**
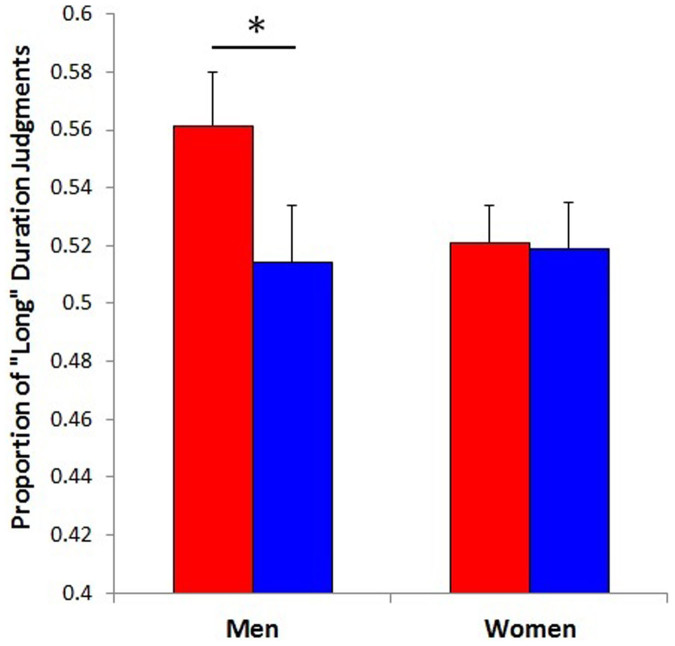
Mean proportion of “long” judgments as a function of the sex-by-color interaction. Asterisk represents a significant difference (* *p* < .001).

**Figure 2:**
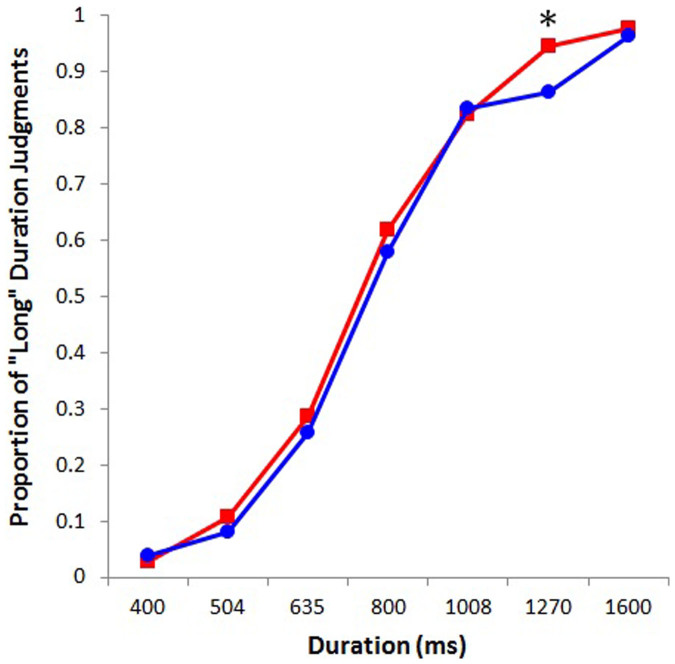
Proportion of “long” judgments plotted against screen durations for red and blue. Asterisk represents a significant difference (* *p* < .001).

An ANOVA treating duration (400, 504, 635, 800, 1008, 1270, and 1600 ms) and color (red or blue) as within-subjects factors and sex (male or female) as a between-subjects factor was performed on the proportion of “long” responses. The results showed a main effect of color, *F*(1, 72) = 14.42, *p* < .001, *η_p_*^*2*^ = .17, indicating that, independent of sex, participants judged the duration of the red screen to be longer than that of the blue screen. In terms of the proportion of participants, 59 percent of all, 43 percent of female, and 76 percent of male participants overestimated the duration of the red screen. We also found a significant interaction between color and sex, *F*(1, 72) = 9.19, *p* < .005, *η_p_*^*2*^ = .11. *Post hoc* analysis (Tukey's test) revealed that the perceived duration of the red screen was longer than that of blue one among men (*p* < .001) but not among women (*p* = .95) (see Fig. 1). Additionally, the ANOVA showed a main effect of duration, *F*(6, 432) = 673.43, *p* < .001, *η_p_*^*2*^ = .90, and an interaction between duration and color, *F*(6, 432) = 5.21, *p* < .001, *η_p_*^*2*^ = .07. Tukey's test revealed that the duration of the red screen was perceived to be longer than that of the blue one at the 1270-ms duration (*p* < .001) (see Fig. 2). None of the other main effects or interactions was significant.

**Figure 3:**
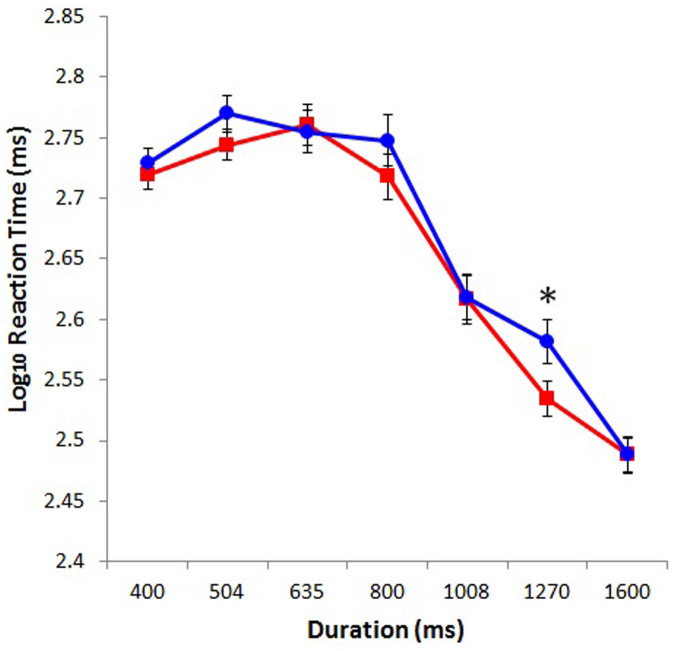
Reaction times plotted against screen durations for red and blue. Asterisk represents a significant difference (* *p* < .05).

Figure 3 shows the reaction times for judging the duration of the blank screen during the testing phase. The reaction times were transformed to a log_10_ scale to improve normality. Reaction times were subjected to ANOVA with duration (400, 504, 635, 800, 1008, 1270, and 1600 ms) and color (red or blue) as a within-subjects factors and sex (male or female) as a between-subjects factor. This ANOVA showed a main effect of color, *F*(1, 72) = 8.98, *p* < .005, *η_p_*^*2*^ = .11, indicating that the perceived duration of the red screen was judged more rapidly than they did the blue one. We also found a main effect of duration, *F*(6, 432) = 127.27, *p* < .001, *η_p_*^*2*^ = .64. Tukey's test revealed that the reaction times for judging the durations of the red and blue screens significantly differed between the 800- and 1008-ms (*p* < .001), between the 1008- and 1270-ms (*p* < .001), and between the 1270- and at 1600-ms conditions (*p* < .001), indicating that the reaction times for judging shorter durations (<1000 ms) were longer than were those for judging longer durations (>1000 ms). Furthermore, the interaction between color and duration was significant, *F*(6, 432) = 2.20, *p* < .05, *η_p_*^*2*^ = .03. Tukey's test revealed that participants judged the duration of the red screen more rapidly than they judged the duration of the blue screen at 1270 ms (*p* < .05) (see Fig. 3). The main effect of sex, *F*(1, 72) = 3.58, *p* = .06, *η_p_*^*2*^ = .05, and the interaction between color and sex, *F*(1, 72) = 1.24, *p* = .27, *η_p_*^*2*^ = .02, were not significant. None of the other interactions was significant, all *F*(6, 432) < 1.08, all *p* > .38.

**Figure 4:**
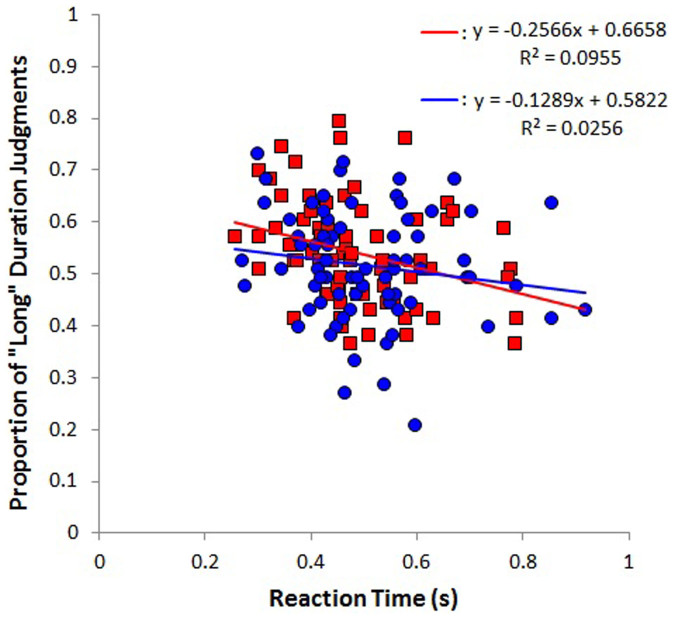
Relationship between reaction times and mean proportion of “long” judgments for red and blue. The red line represents an approximation of the red trials; the blue line represents that for the blue trials.

Figure 4 shows the relationship between reaction times for judging the duration of a screen of each color and the proportion of long responses. Pearson's correlations were used to assess these relationships. We found a significant correlation between reaction time and the proportion of long responses when the screen was red (*r* = −.31, *t*(72) = 2.76, *p* < .01), indicating that more rapid responses were associated with longer perceived durations under the red-screen condition. However, no such correlation was evident when the screen was blue (*r* = −.16, *t* (72) = 1.37, *p* = .17).

## Discussion

The present study showed that the duration of the red screen was perceived as longer than was that of the blue screen by male participants. Additionally, the reaction times for judging the duration of the red screen were faster than were those of the blue screen among both men and women.

These results are consistent with those of previous studies indicating that red induces higher arousal levels than does blue^1^,^2^, and that the average reaction time to red is faster than that to blue^9^,^10^,^11^,^12^. Previous studies have shown that observing emotional expressions affects the subjective experience of time (i.e., overestimation) by increasing the level of arousal of the perceiver^3^,^4^,^5^. For the same reason, it is plausible that the arousal induced by red increases the speed of the internal clock, resulting in the overestimation of the duration of a red screen.

The present study also showed that the reaction times of both men and women for judging the duration of the red screen were shorter, which also seems to reflect the arousal effect of red. Although we found no significant sex differences in the arousal effect, female participants did not overestimate the duration of red screen. Recent studies have shown that time perception cannot be reduced to arousal because emotional valence also plays a critical role in this phenomenon^13^,^14^,^15^. If we take emotional valence into consideration, the sex difference in the perceived duration of the red screen may derive not from a sex difference in arousal levels but from a sex difference in the emotional valence of red. Indeed, some studies have found that the effect of red is sex dependent. For example, Hill and Barton^16^ reported that contestants wearing red were more likely to win in four combat sports (boxing, taekwondo, Greco–Roman wrestling, and freestyle wrestling) in the 2004 Athens Olympics. However, a subsequent analysis of the Olympics data found that the red-associated winning bias was only apparent in men^17^. Additionally, Ioan et al.^18^ investigated the distractor effect of red during a computerized Stroop task. Participants were presented with a series of colored word stimuli and were asked to name the color in which the words were written while disregarding the actual meaning of the words. In general, the reaction times to incongruent stimuli (e.g., “BLUE” printed in red ink) were longer than were those to congruent stimuli (e.g., “BLUE” printed in blue ink). This Stroop interference effect was more pronounced in men than in women when red color names were used. Moreover, a red progress bar impeded performance in web-based tests of general knowledge only for male participants^19^.

Red is a sign of dominance in non-human primates^20^,^21^, and human anger is associated with a reddening of the skin due to increased blood flow^22^,^23^. For this reason, sexual selection may have influenced the evolution of the human response to red in competition^18^. The males of many species are biologically programmed to compete with other males for status, territory, mates, and so on, and sensitivity to the meaning of red as a sign of dominance may be helpful in judgments about potential physical conflicts with opponents^24^. In contrast, females are inherently less competitive, a phenomenon that has been attributed to differential reproductive costs for men and women^25^, rendering the association between red and dominance of less importance for survival among women than among men^19^. According to this reasoning, the emotional valence of red may differ for males and females. The perceived duration of pictures with different emotional valences differed even when the arousal level associated with those pictures was equal^13^,^14^,^15^. For that reason, the difference between males and females in the perceived duration of the red screen may be attributable to sex differences in the emotional valence of red.

It is a little puzzling why the color effect appeared so outstanding in the condition of 1270-ms. Another experiment with children using the same program and procedure did not show such a bias (unpublished). Even though we examined the color effect without data in condition of 1270-ms, the main effect of color, *F* (1, 72) = 4.09, *p* < .05, *η_p_*^*2*^ = .054, and the interaction between color and sex, *F* (1, 72) = 7.64, *p* < .01, *η_p_*^*2*^ = .096, were significant, indicating that the color effect was not restricted in the condition of 1270-ms.

The mechanisms of temporal processing are different between sub- and supra-second intervals^26^,^27^. Though memory systems such as working memory are important for temporal processing of supra-second intervals^28^, timing of sub-second intervals is assumed to be relatively automatic process and beyond cognitive control^29^. Unlike our study using intervals in the milliseconds range up to 1.6 s, previous studies involving the relation between color and time perception have used multiple seconds and those results might be influenced by cognitive control. It is possible that color influence is also varied according to time intervals. The further study using other time intervals would be necessary to generalize our findings.

The present study showed that red increased perceived duration and that the duration of the red screen was overestimated by participants who responded to red quickly. However, only male participants overestimated the duration of the red screen. Some studies indicate that women's sensitivity to red is associated with menstrual cycles^18^,^30^, and the variability in menstrual cycles may have contributed to our results regarding perceptions by female participants of the duration of the red screen. The present study presented the color red in the context of tests about time estimation, and the influence of color on psychological functioning varies as a function of the psychological context in which color is perceived^31^. For example, in the context of price perceptions, prices were evaluated more favorably when they appeared in red than when they appeared in black. However, this effect occurred in men, but not in women^32^. Sex differences in the effect of red may vary across contexts, and future studies should consider the effect of red on time perception in various contexts.

## Methods

This study was conducted according to the Declaration of Helsinki and the ethical guidelines for experiments with human participants.

### Participants

The participants consisted of 74 undergraduate students: 37 men (mean age = 21.92 years; *SD* = 0.43) and 37 women (mean age = 19.81 years, *SD* = 0.78). Each received course credit in return for their participation. All the participants gave written informed consent to participate in this study, which was approved by the Ethical Committee of the Primate Research Institute of Kyoto University (H2012-12).

### Materials

A computer with a color monitor controlled experimental events. The experiment was programmed and run using Visual Basic 6.0. The participants responded by clicking one of three mouse buttons (right, left, or middle). We presented white rectangles (6 × 7 cm) on the computer screen during the training and testing phases. During the training phase, feedback for correct responses was provided in the form of a chime, and that for incorrect responses was provided in the form of a buzzer. During the testing phase, the whole screen color temporarily turned red (#FF0000; hue = 0, saturation = 100, Luminosity = 100) or blue (#0000FF; hue = 240, saturation = 100, Luminosity = 100) as colored stimuli. However, this study did not use a spectrophotometer to manipulate the color of screen. Therefore, displayed color might differ from the color specified by the three dimensions (hue, saturation, and luminosity).

### Procedure

During the training phase, each trial was initiated by the presentation of a white rectangle at the center of a black screen. When the participant clicked the middle mouse button, the stimulus disappeared, and two white rectangles appeared side by side. In half the trials, the rectangles appeared after 400 ms (short). In the remaining trials, the rectangles appeared after 1600 ms (long). Participants were trained to click the left and right mouse buttons under the short or long condition, respectively (click buttons were counterbalanced across participants). When a participant clicked the correct button, a chime sounded; an incorrect response was followed by a buzzer sound. After the response, the rectangles disappeared, and an inter-trial interval (ITI) of random duration between 1 and 2 s followed. Each participant completed 20 trials in random order (10 short and 10 long). Training terminated when the participant made no errors during the eight consecutive trials. If the participant failed to reach the criterion, 20 additional trials were conducted. During the testing phase, five intermediate-duration intervals (504, 635, 800, 1008, and 1270 ms), equally spaced in logarithmic terms, were introduced in addition to the two standard intervals (400 and 1600 ms). As in the training phase, the participants were required to indicate whether the duration of the blank screen was similar to the short or to the long standard duration, but no feedback was given. During this phase, the color of the blank screen was changed from black to red (#FF0000) or blue (#0000FF). Participants were given 126 trials (i.e., each of the two colors, red and blue, was presented nine times, once for each of the seven durations (two anchors and five probes)). The seven durations and two colored screens were quasi-randomly assigned, and the only restriction was that each duration and each color appear no more than three times in a row.

## Author Contributions

M.S. designed and conducted the experiments as well as wrote the paper. M.S. and N.M. read and approved the final manuscript.

## References

[b1] Ali M. R. Pattern of EEG recovery under photic stimulation by light of different colors. Electroencephalogr Clin Neurophysiol. 33, 332–335 (1972). 4114919 10.1016/0013-4694(72)90162-9

[b2] Jacobs K. W. & Hustmyer F. E. Jr Effects of four psychological primary colors on GSR, heart rate and respiration rate. Percept Mot Skills. 38, 763–766 (1974). 4842431 10.2466/pms.1974.38.3.763

[b3] Effron D. A., Niedenthal P. M., Gil S. & Droit-Volet S. Embodied temporal perception of emotion. Emotion. 6, 1–9 (2006). 16637745 10.1037/1528-3542.6.1.1

[b4] Gil S., Niedenthal P. M. & Droit-Volet S. Anger and time perception in children. Emotion. 7, 219–225 (2007). 17352578 10.1037/1528-3542.7.1.219

[b5] Tipples J. When time stands still: fear-specific modulation of temporal bias due to threat. Emotion. 11, 74–80 (2011). 21401227 10.1037/a0022015

[b6] Smets G. Time expression of red and blue. Percept Mot Skills. 29, 511–514 (1969). 5361716 10.2466/pms.1969.29.2.511

[b7] Caldwell J. A. & Jones G. E. The effects of exposure to red and blue light on physiological indices and time estimation. Perception. 14, 19–29 (1985). 4069932 10.1068/p140019

[b8] Gorn G. J., Chattopadhyay A., Sengupta J. & Tripathi S. Waiting for the web: how screen color affects time perception. J. Mark. Res. 41, 215–225 (2004).

[b9] Elliot A. J. & Aarts H. Perception of the color red enhances the force and velocity of motor output. Emotion. 11, 445–449 (2011). 21500913 10.1037/a0022599

[b10] Guéguen N., Jacob C., Lourel M. & Pascual A. When drivers see red: car color frustrators and drivers' aggressiveness. Aggress Behav. 38, 166–169 (2012). 10.1002/ab.21416 25363640

[b11] Pieron H. The sensations, their functions, processes, and mechanisms. (Yale University Press, 1952).

[b12] Pollack J. D. Reaction time to different wavelengths at various luminances. Percept. Psychophys. 3, 17–24 (1968).

[b13] Angrilli A., Cherubini P., Pavese A. & Manfredini S. The influence of affective factors on time perception. Percept. Psychophys. 59, 972–982 (1997). 9270369 10.3758/bf03205512

[b14] Gil S. & Droit-Volet S. Emotional time distortions: the fundamental role of arousal. Cogn Emot. 26, 847–862 (2012). 22296278 10.1080/02699931.2011.625401

[b15] Smith S. D., McIver T. A., Di Nella M. S. J. & Crease M. L. The effects of valence and arousal on the emotional modulation of time perception: evidence for multiple stages of processing. Emotion. 11, 1305–1313 (2011). 22142208 10.1037/a0026145

[b16] Hill R. A. & Barton R. A. Psychology: red enhances human performance in contests. Nature. 435, 293–293 (2005). 15902246 10.1038/435293a

[b17] Barton R. A. & Hill R. A. Sporting contests: Seeing red? Putting sportswear in context (reply). Nature. 437, E10–E11 (2005). 16251904 10.1038/nature04306

[b18] Ioan S. *et al.* Red is a distractor for men in competition. Evol Human Behav. 28, 285–293 (2007).

[b19] Gnambs T., Appel M. & Batinic B. Color red in web-based knowledge testing. Comput Hum Behav. 26, 1625–1631 (2010).

[b20] Setchell J. M., Charpentier M. & Wickings E. J. Mate guarding and paternity in mandrills: factors influencing alpha male monopoly. Anim Behav. 70, 1105–1120 (2005).

[b21] Waitt C., Gerald M. S., Little A. C. & Kraiselburd E. Selective attention toward female secondary sexual color in male rhesus macaques. Am. J. Primatol. 68, 738–744 (2006). 16786524 10.1002/ajp.20264

[b22] Changizi M. A., Zhang Q. & Shimojo S. Bare skin, blood and the evolution of primate colour vision. Biol. Lett. 2, 217–221 (2006). 17148366 10.1098/rsbl.2006.0440 PMC1618887

[b23] Fetterman A. K., Robinson M. D., Gordon R. D. & Elliot A. J. Anger as seeing red perceptual sources of evidence. Soc Psychol Pers Sci. 2, 311–316 (2011). 10.1177/1948550610390051 PMC3399410 22822418

[b24] Little A. C. & Hill R. A. Attribution to red suggests special role in dominance signalling. J. Evol. Psychol. 5, 161–168 (2007).

[b25] Buss D. M. Evolutionary psychology: the new science of the mind. (Pearson, 2012).

[b26] Lewis P. A. & Miall R. C. Brain activation patterns during measurement of sub-and supra-second intervals. Neuropsychologia. 41, 1583–1592 (2003). 12887983 10.1016/s0028-3932(03)00118-0

[b27] Näätänen R., Syssoeva O. & Takegata R. Automatic time perception in the human brain for intervals ranging from milliseconds to seconds. Psychophysiology. 41, 660–663 (2004). 15189489 10.1111/j.1469-8986.2004.00182.x

[b28] Lee K.-H. *et al.* Time perception and its neuropsychological correlates in patients with schizophrenia and in healthy volunteers. Psychiat. Res. 166, 174–183 (2009). 10.1016/j.psychres.2008.03.004 19278734

[b29] Smith J. G., Harper D. N., Gittings D. & Abernethy D. The effect of Parkinson's disease on time estimation as a function of stimulus duration range and modality. Brain Cognition. 64, 130–143 (2007). 17343966 10.1016/j.bandc.2007.01.005

[b30] Beall A. T. & Tracy J. L. Women are more likely to wear red or pink at peak fertility. Psychol. Sci. 24, 1837–1841 (2013). 23842955 10.1177/0956797613476045

[b31] Meier B. P., D'Agostino P. R., Elliot A. J., Maier M. A. & Wilkowski B. M. Color in context: Psychological context moderates the influence of red on approach-and avoidance-motivated behavior. PloS One. 7, e40333 (2012). 22808136 10.1371/journal.pone.0040333 PMC3394796

[b32] Puccinelli N. M., Chandrashekaran R., Grewal D. & Suri R. Are men seduced by red? The effect of red versus black prices on price perceptions. J. Retailing. 89, 115–125 (2013).

